# Whole Exome Sequencing in Atrial Fibrillation

**DOI:** 10.1371/journal.pgen.1006284

**Published:** 2016-09-02

**Authors:** Steven A. Lubitz, Jennifer A. Brody, Nathan A. Bihlmeyer, Carolina Roselli, Lu-Chen Weng, Ingrid E. Christophersen, Alvaro Alonso, Eric Boerwinkle, Richard A. Gibbs, Joshua C. Bis, NHLBI GO Exome Sequencing Project, L. Adrienne Cupples, Peter J. Mohler, Deborah A. Nickerson, Donna Muzny, Marco V. Perez, Bruce M. Psaty, Elsayed Z. Soliman, Nona Sotoodehnia, Kathryn L. Lunetta, Emelia J. Benjamin, Susan R. Heckbert, Dan E. Arking, Patrick T. Ellinor, Honghuang Lin

**Affiliations:** 1 Cardiac Arrhythmia Service, Massachusetts General Hospital, Boston, Massachusetts, United States of America; 2 Program in Medical and Population Genetics, The Broad Insitute of Harvard and MIT, Cambridge, Massachusetts, United States of America; 3 Cardiovascular Health Research Unit, Department of Medicine, University of Washington, Seattle, Washington, United States of America; 4 McKusick-Nathans Institute of Genetic Medicine, Johns Hopkins University School of Medicine, Baltimore, Maryland, United States of America; 5 Cardiovascular Research Center, Massachusetts General Hospital, Boston, Massachusetts, United States of America; 6 Department of Medical Research, Bærum Hospital, Vestre Viken Hospital Trust, Bærum, Norway; 7 Department of Epidemiology, Rollins School of Public Health, Emory University, Atlanta, Georgia, United States of America; 8 Human Genetics Center, UTHealth, Houston, Texas, United States of America; 9 Human Genome Sequencing Center, Baylor College of Medicine, Houston, Texas, United States of America; 10 Department of Biostatistics, Boston University School of Public Health, Boston, Massachusetts, United States of America; 11 Boston University and National Heart, Lung and Blood Institute’s Framingham Heart Study, Framingham, Massachusetts, United States of America; 12 Dorothy M. Davis Heart & Lung Research Institute, The Ohio State University Wexner Medical Center, Columbus, Ohio, United States of America; 13 Department of Genome Sciences, University of Washington, Seattle, Washington, United States of America; 14 Division of Cardiovascular Medicine, Department of Medicine, Stanford University School of Medicine, Stanford, California, United States of America; 15 Cardiovascular Health Research Unit, Departments of Medicine, Epidemiology, and Health Services, University of Washington, Seattle, Washington, United States of America; 16 Group Health Research Institute, Group Health Cooperative, Seattle, Washington, United States of America; 17 Epidemiological Cardiology Research Center (EPICARE), Wake Forest School of Medicine, Winston-Salem, North Carolina, United States of America; 18 Division of Cardiology, University of Washington, Seattle, Washington, United States of America; 19 Section of Cardiovascular Medicine, Department of Medicine, Boston University School of Medicine, Boston, Massachusetts, United States of America; 20 Department of Epidemiology, School of Public Health, Boston University, Boston, Massachusetts, United States of America; 21 Preventive Medicine Section, Department of Medicine, Boston University School of Medicine, Boston, Massachusetts, United States of America; 22 Department of Epidemiology, University of Washington, Seattle, Washington, United States of America; 23 Section of Computational Biomedicine, Department of Medicine, Boston University School of Medicine, Boston, Massachusetts, United States of America; Mount Sinai School of Medicine, UNITED STATES

## Abstract

Atrial fibrillation (AF) is a morbid and heritable arrhythmia. Over 35 genes have been reported to underlie AF, most of which were described in small candidate gene association studies. Replication remains lacking for most, and therefore the contribution of coding variation to AF susceptibility remains poorly understood. We examined whole exome sequencing data in a large community-based sample of 1,734 individuals with and 9,423 without AF from the Framingham Heart Study, Cardiovascular Health Study, Atherosclerosis Risk in Communities Study, and NHLBI-GO Exome Sequencing Project and meta-analyzed the results. We also examined whether genetic variation was enriched in suspected AF genes (N = 37) in AF cases versus controls. The mean age ranged from 59 to 73 years; 8,656 (78%) were of European ancestry. None of the 99,404 common variants evaluated was significantly associated after adjusting for multiple testing. Among the most significantly associated variants was a common (allele frequency = 86%) missense variant in *SYNPO2L* (rs3812629, p.Pro707Leu, [odds ratio 1.27, 95% confidence interval 1.13–1.43, *P* = 6.6x10^-5^]) which lies at a known AF susceptibility locus and is in linkage disequilibrium with a top marker from prior analyses at the locus. We did not observe significant associations between rare variants and AF in gene-based tests. Individuals with AF did not display any statistically significant enrichment for common or rare coding variation in previously implicated AF genes. In conclusion, we did not observe associations between coding genetic variants and AF, suggesting that large-effect coding variation is not the predominant mechanism underlying AF. A coding variant in *SYNPO2L* requires further evaluation to determine whether it is causally related to AF. Efforts to identify biologically meaningful coding variation underlying AF may require large sample sizes or populations enriched for large genetic effects.

## Author Summary

Atrial fibrillation is a common and morbid cardiac arrhythmia. Atrial fibrillation is heritable, and numerous genome-wide susceptibility loci have been identified, predominantly in non-coding regions. Over 35 genes also have been implicated in atrial fibrillation pathogenesis mostly through prior smaller scale candidate gene association studies, which generally did not have robust replication to support the associations. Therefore, the role of coding variation in the biology of atrial fibrillation is unclear. We examined whole exome sequencing data from 1,734 individuals with and 9,423 without atrial fibrillation, and did not observe any significant associations between coding variation and the arrhythmia. Furthermore, we did not observe any enrichment for association in previously implicated atrial fibrillation genes. In aggregate, our findings suggest that large effect coding variation is unlikely to be a predominant mechanism of common forms of atrial fibrillation encountered in the community.

## Introduction

Atrial fibrillation (AF) is a common [1, 2] arrhythmia associated with substantial morbidity [3–7]. Current treatments for AF have limited efficacy and can cause significant adverse effects [8, 9]. AF is heritable and approximately one in four individuals with AF has a first-degree relative with the condition [10].

In recent years a large number of genes have been implicated in AF risk using both genome-wide association studies and candidate gene screening approaches. Large-scale genome-wide association studies have identified multiple AF susceptibility loci [11–15], and the top variants at discovered loci have largely been localized to noncoding regions of the genome. In contrast, there have been over 35 genes implicated in AF in candidate gene studies [16]. These studies have had a number of limitations including small sample sizes, consideration of only one or a small number of genes, and the lack of suitable control populations. To date, large-scale studies to determine whether these genes are truly related to AF have not been performed.

Since the discovery of genes causally related to AF may enable a better understanding of AF pathogenesis and potentially inform the development of therapies for AF, there is a critical need to systematically identify the genetic basis of AF. We therefore sought to assess the relations between coding variation and AF in a large sample of individuals who underwent whole exome sequencing. We further sought to determine whether coding variation in genes implicated in AF was enriched among AF cases.

## Results

The current analysis included 6,737 participants of European ancestry (n = 1,155 AF events) and 1,246 participants of African ancestry (n = 246 AF cases) from a Cohorts for Heart and Aging Research in Genomic Epidemiology (CHARGE) exome sequencing effort and 1,919 participants of European ancestry (n = 233 cases) and 1,255 participants of African ancestry (n = 100 AF events) from the NHLBI-GO Exome Sequencing Project (ESP). The clinical characteristics of studied participants are listed in Table 1. Sequencing coverage for the subset of AF genes is provided in S2 Table.

**Table 1:** Baseline characteristics of the participating studies.

Ancestry	Study	Type of Sample	Number of Samples	Age, y	Men	Hypertension	Heart failure
European	ARIC	Case	730	69.9 ± 7.3	451 (61.8%)	305 (41.8%)	54 (7.4%)
		Referent	4,000	73.5 ± 6.3	1819 (45.5%)	942 (23.5%)	105 (2.6%)
	CHS	Case	251	73.5 ± 5.7	122 (48.6%)	148 (59.0%)	0
		Referent	500	72.6 ± 5.7	233 (46.6%)	274 (54.8%)	0
	ESP	Case	233	65.0 ± 10.3	118 (50.6%)	132 (56.7%)	NA
		Referent	1,686	61.3 ± 9.0	396 (23.5%)	716 (42.5%)	NA
	FHS	Case	174	69.9 ± 9.0	107 (61.5%)	115 (66.1%)	13 (7.5%)
		Referent	1,082	59.0 ± 11.2	478 (44.2%)	418 (38.6%)	6 (0.6%)
African	ARIC	Case	246	69.4 ± 7.3	107 (43.5%)	163 (66.3%)	25 (10.2%)
		Referent	1,000	71.3 ± 6.5	345 (34.5%)	507 (50.7%)	50 (5%)
	ESP	Case	100	63.1 ± 8.7	14 (14.0%)	63 (63.0%)	NA
		Referent	1,155	59.4 ± 7.7	161 (13.9%)	616 (53.3%)	NA
***Total***		Case	***1*,*734***				
		Referent	***9*,*423***				

Data displayed as mean ± standard deviation or number (%). ARIC = Atherosclerosis Risk in Communities Study; CHS = Cardiovascular Health Study; FHS = Framingham Heart Study; ESP = NHLBI-GO Exome Sequencing Project. NA = not available.

### Association of common variants with AF

**Fig 1:**
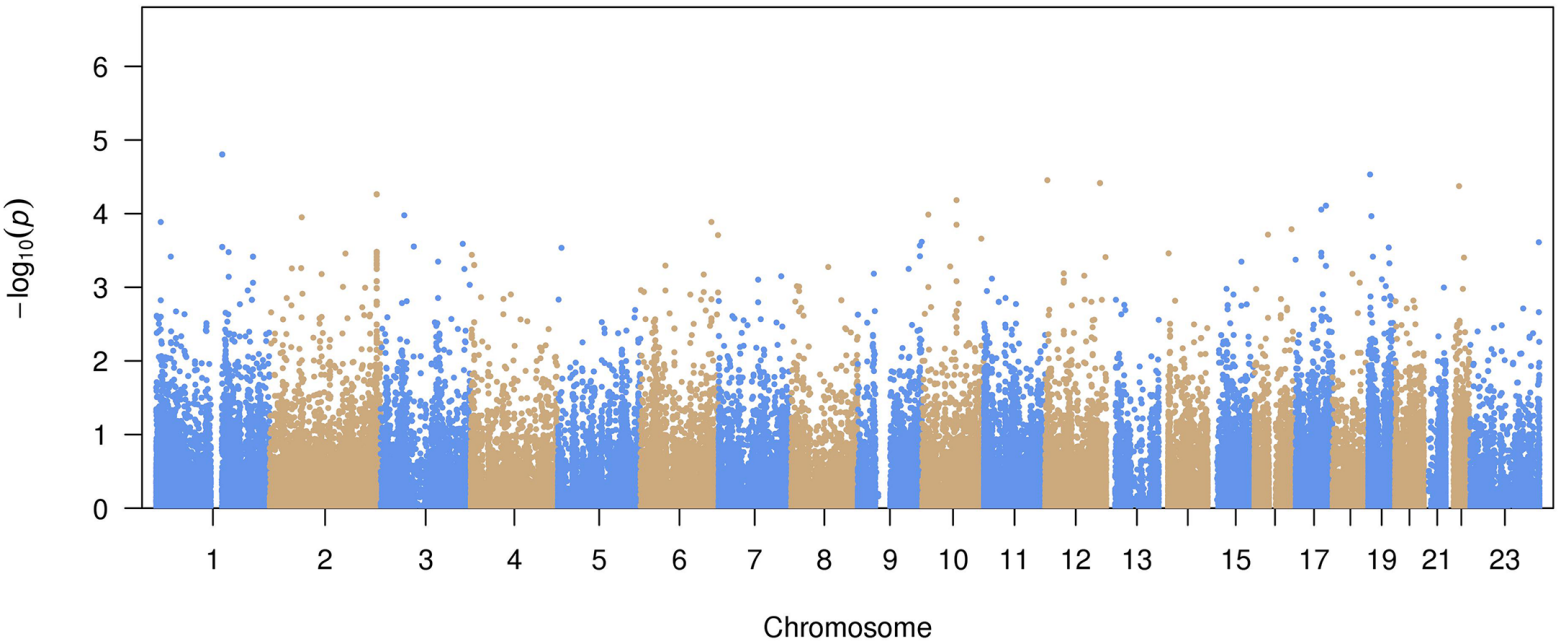
Manhattan plot of common variant associations with atrial fibrillation.

A total of 99,404 common variants (MAF≥0.01) were included in our study. Approximately 99.7% of the variants were already reported in dbSNP (version 142) or the 1000 Genomes Project. The Manhattan plot representing the primary pooled ancestry analysis is displayed in Fig 1 and the QQ plot is shown in S1 Fig. No inflation of Type I error was observed (genomic control λ = 0.91).

The top 15 variants most significantly associated with AF are listed in Table 2. No common variants were significantly associated with AF after Bonferroni correction for multiple testing (all *P*>0.05/99,404 = 5.0x10^-7^). The most significantly associated variant was rs56025621 (*P* = 1.6x10^-5^), which is located in the first intron of *HFE2*, a gene encoding the hemochromatosis type 2 peptide. The SNP was not genotyped in HapMap phase II, thus the association between rs56025621 and AF was not assessed in previous genome-wide association studies.

**Table 2:** Most significant common variants associated with atrial fibrillation.

SNP	Locus	Gene	Risk allele	Non-risk allele	Risk allele frequency	Function	Odds ratio	*P*-value
							(95% CI)	
rs56025621	1q21.1	*HFE2*	C	G	0.98	Intronic	1.89 (1.41–2.52)	1.6x10^-5^
rs115664635	19p13.3	*EBI3*	T	C	0.01	Intronic	2.15 (1.50–3.09)	2.9x10^-5^
rs61635955	12p13.32	*KCNA6*	G	A	0.01	UTR3	2.31 (1.55–3.43)	3.5 x10^-5^
rs74614893	12q24.23	*CIT*	A	G	0.02	Intronic	2.48 (1.61–3.82)	3.8 x10^-5^
rs1805129	22q12.1	*CHEK2*	C	T	0.03	Synonymous	1.59 (1.28–1.99)	4.2 x10^-5^
rs6759892	2q37.1	*UGT1A9*	G	T	0.41	Intronic	1.30 (1.15–1.48)	5.5x10^-5^
rs3812629	10q22.2	*SYNPO2L*	G	A	0.86	Missense	1.27 (1.13–1.43)	6.6x10^-5^
rs2286562	17q24.2	*FAM20A*	C	T	0.83	Synonymous	1.24 (1.12–1.39)	7.8x10^-5^
rs8178318	17q22	*LPO*	T	C	0.01	Missense	2.00 (1.41–2.82)	8.8x10^-5^
rs11258476	10p13	*PRPF18*	G	A	0.98	Synonymous	1.72 (1.31–2.27)	1.0x10^-4^
rs114209632	3p21.2	*POC1A*	T	C	0.08	Intronic	1.36 (1.17–1.59)	1.1x10^-4^
rs2962	19p13.2	*INSR*	G	A	0.92	Synonymous	1.34 (1.15–1.55)	1.1x10^-4^
rs12989348	2p13.3	*C2orf42*	G	A	0.88	Synonymous	1.29 (1.13–1.46)	1.1x10^-4^
rs11751128	6q25.3	*TIAM2*	T	C	0.24	Missense	1.22 (1.10–1.35)	1.3x10^-4^
rs778228	1p36.22	*CASZ1*	A	G	0.77	Synonymous	1.21 (1.10–1.33)	1.3x10^-4^

CI: confidence interval

The SNP rs3812629, a missense variant encoding a proline to leucine amino acid substitution at amino acid 707 of *SYNPO2L*, occurs at a genome-wide significant disease susceptibility locus for AF [15]. The variant is in moderate linkage disequilibrium (r^2^ = 0.69 European ancestry, 1000 Genomes Project) with the top SNP (rs10824026) associated with AF at the locus in a prior genome-wide association study [15]. In the subset of individuals with both genome-wide genotyping data and exome sequence data available from ARIC (n = 6,630), CHS (n = 671), and FHS (n = 1,256) we examined associations between the top noncoding SNP (rs10824026) and the p.Pro707Leu (rs3812629) variant with AF after adjustment for one another (S3 Table). Adjustment for the coding variant attenuated the signal of the lead GWAS SNP in the analysis, and vice versa, suggesting the two variants represent the same AF susceptibility signal.

Previously reported top SNPs for AF derived from genome-wide association studies [15, 17], which are located in noncoding regions, were not assayed using the capture arrays in this study. As such, they were not analyzed in the current analysis.

### Association of rare variants with AF

We collapsed rare variants (MAF<1%) into gene regions and performed association testing between each gene region with AF. Our primary analysis was restricted to nonsynonymous and splice-site variants. We excluded gene regions with a cumulative MAF less than 1%. In total, we tested 8,879 gene regions. None of the gene regions were significantly associated with AF after adjusting for multiple testing (all p>0.05/8,879 = 5.6x10^-6^). The most significantly associated gene region was *IL17REL* (p = 1.3x10^-5^), a gene encoding interleukin 17 receptor E-like (S4 Table). The most significant single variant in *IL17REL* in this analysis was rs200958270 (OR 6.92, 95% CI 3.38–14.15, p = 1.2x10^-7^), a missense (p.Glu151Gly) variant that has a minor allele frequency of 0.004%. The variant did not meet our prespecified significance criteria for association. In a secondary analysis restricted to damaging variants, no specific gene regions were significantly associated with AF (S5 Table). Again, the most significantly associated gene in the damaging analysis was again *IL17REL* (p = 1.9x10^-6^). Variants in *IL17REL* have been implicated in inflammatory bowel disease [18, 19] though the relations between variation in *IL17REL* and cardiac function are unclear.

We also examined the associations between rare coding variants and AF within reported AF-susceptibility genes (Table 3). None were significantly associated with AF after adjusting for multiple testing.

**Table 3:** Genes previously implicated in atrial fibrillation pathogenesis.

Gene	SKAT P-value	No. variants	Gene	SKAT P-value	No. variants
*ACE*	0.63	150	KCNN3*	–	0
*AGT*	0.53	60	*KCNQ1*	0.61	56
*ANK2*	0.18	292	*LMNA*	0.35	47
C9ORF3*	0.73	66	MYOZ1*	–	0
CAND2*	0.59	105	NEURL*	–	0
CAV1*	–	0	*NPPA*	–	0
*CAV3*	–	0	*NUP155*	0.03	79
*GATA4*	0.37	26	PITX2*	–	0
*GATA5*	0.37	29	PRRX1*	0.98	19
*GATA6*	–	0	*SCN10A*	0.77	180
GJA1*	–	0	*SCN1B*	–	0
*GJA5*	–	0	*SCN2B*	–	0
HCN4*	0.32	56	*SCN3B*	–	0
IL6R*	–	0	*SCN5A*	0.51	149
*KCNA5*	0.23	49	SYNE2*	0.42	591
*KCNE1*	–	0	SYNPO2L*	0.96	69
*KCNE2*	–	0	TBX5*	0.51	43
*KCNH2*	–	0	ZFHX3*	0.55	319
*KCNJ2*	–	0			

Variants include nonsynonymous and splice-site variants with allele frequencies <1%.

*Genes implicated by large-scale discovery efforts in individuals of European ancestry. References for genes are provided in the online supplement.

In a post-hoc exploratory analyses, we included all rare variants (<1%) within each gene region, irrespective of annotation, and tested them for association with AF using an adjusted significance threshold of p = 2.5x10^-6^ (0.05/19913 genes). The results are summarized in S6 Table. The lead gene associated with AF was *ACY3* (p = 2.2x10^-7^), which encodes aminoacylase 3. No relation between ACY3 and cardiac function or arrhythmias has been described previously.

**Fig 2:**
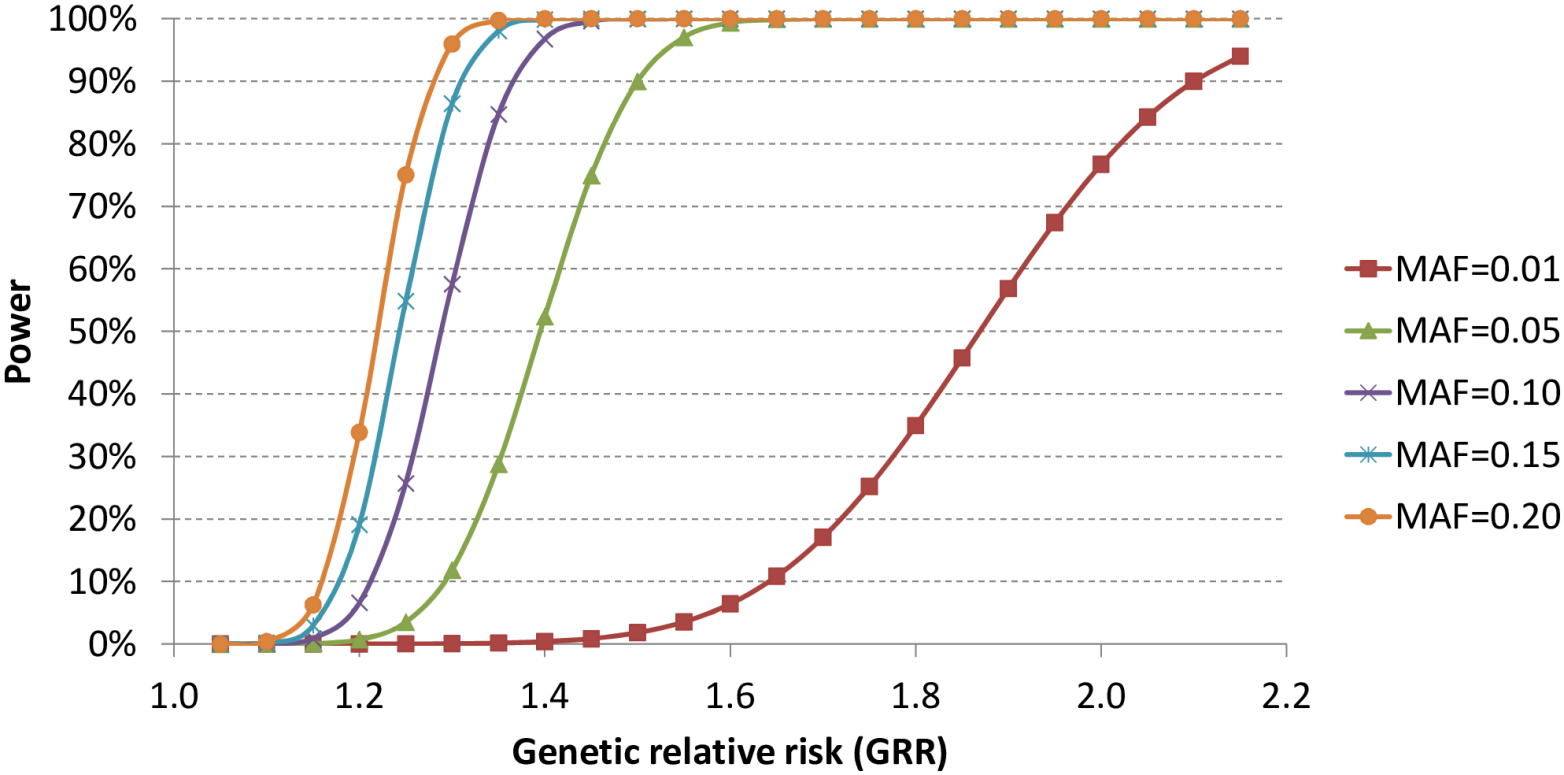
Statistical power with current sample size and α = 5x10^-7^.

With the current sample size, we estimated the statistical power to identify genetic variants with α = 5x10^-7^, assuming 100,000 independent tests. As shown in Fig 2, we had limited statistical power to identify genetic variants with allele frequencies as low as 1% unless the genetic relative risk was higher than two. In contrast, the statistical power increased significantly for relatively common variants with allele frequencies of at least 5%.

### Pathway enrichment analysis

We subsequently assessed whether genetic variation in pre-specified gene sets was enriched among individuals with AF. We did not observe enrichment for common (FDR = 0.38) or rare (FDR = 0.91) variation in reported AF-related genes among individuals with AF (Table 4).

**Table 4:** Assessment of variant enrichment in genes purportedly implicated in atrial fibrillation.

Gene set	Common or rare variants	No. genes with variants eligible for analysis	Normalized effect size	*P*-value	False discovery rate q-value
Atrial fibrillation (No. = 37 genes)					
	Common	37	1.12	0.28	0.38
	Rare	19	0.75	0.87	0.91

Rare variants were defined as those with minor allele frequencies < 1%, and common as those with frequencies ≥ 1%.

## Discussion

In our sample of 1,734 individuals with and 9,423 without AF who underwent whole exome sequencing, we did not observe any rare coding variation significantly associated with AF. Our observations suggest that coding variation with large effect sizes is unlikely to be the predominant mechanism underlying common forms of AF.

Our results extend prior literature focusing on coding variation underlying AF. Numerous reports propose coding variation as a mechanism underlying AF (S1 Table). However, much of the prior literature was generated via candidate gene association studies. Such discoveries have not been routinely replicated, and the studies were of small size, potentially favoring spurious results. Indeed, we previously observed that most findings from prior AF candidate gene association studies were not replicated when tested in additional study samples [17].

In the context of prior literature and our sample size, our study has two major implications for understanding AF pathogenesis. First, the lack of observed association between coding variation and AF implies that large effect coding variation is not likely to be common in typical forms of AF. In contrast, both noncoding variation, and coding variation with smaller effect sizes, may contribute to AF pathogenesis. Genome-wide association studies have identified highly associated common genetic variants near ion channels, cardiac and pulmonary transcription factors, and other genes in individuals with AF [11–15], underscoring the polygenic nature of AF. Nevertheless, the causal variants and genes underlying the arrhythmia remain unknown. Future whole-genome sequencing efforts may help to clarify the genetic contributions to AF.

Second, our findings suggest that efforts to identify potential therapeutic targets for AF through exome sequencing analyses will require much larger sample sizes or populations enriched for large genetic effects. Such populations might include those with early onset AF or consanguineous populations with the propensity to homozygous loss of function alleles in genes. Nevertheless, the additional cost required to sequence such large populations must be balanced against the potentially more cost-efficient approach of performing GWAS genotyping, imputation, and subsequent functional characterization for genetic discovery. The lack of observation of any prominent coding variation underlying AF is consistent with other whole exome sequencing efforts of complex diseases such as coronary disease and diabetes [20], which generally have not identified coding variation as the major mechanisms underlying these conditions.

Our study should be interpreted in the context of the study design. Our study was predominantly comprised of individuals of European ancestry, and therefore the findings may not be generalizable to other ancestral groups. The individuals with AF may have had multiple etiologies for the condition, and may not have been enriched for genetic forms of the arrhythmia. We cannot exclude that AF may have been misclassified, especially since AF may be paroxysmal and asymptomatic at times. Such misclassification is expected to bias the results toward the null. Furthermore, our study had limited power to assess the role of many coding variants, particularly because classifying missense variants as pathogenic or not remains challenging despite the routine use of bioinformatic algorithms. An earlier report of whole exome sequencing in 6 families with AF has summarized some of the bioinformatics challenges of utilizing whole exome sequencing data [21]. The size of our study sample limited our ability to detect potentially functional rare variants. Additionally, we utilized a Bonferroni significance threshold, which may be overly conservative for genetic discovery.

In conclusion, we observed that coding variation is not a major contributor to AF in a sample of individuals predominantly of European ancestry. Efforts to identify coding variation underlying AF will require much larger study samples. Future analyses that integrate coding and noncoding variation, such as whole genome sequencing, are warranted.

## Materials and Methods

### Study participants

The current study included participants from three population-based cohorts that participated in the CHARGE exome sequencing effort (N = 15,459 individuals of either European or African ancestry): the Atherosclerosis Risk in Communities study (ARIC), Cardiovascular Health Study (CHS), and Framingham Heart Study (FHS). In ARIC, a random subset of 4000 European ancestry control subjects and 1000 African ancestry subjects were chosen without regard for age or sex matching. Each cohort has been described in detail previously [22–25].

We also included individuals from ESP (N = 6823 individuals of European or African ancestry) in whom AF data were ascertained (cohorts included ARIC, CHS, FHS and the Women's Health Initiative) [26]. We omitted from analysis samples for whom phenotypic data for AF were missing (N = 2593 CHARGE, N = 3689 ESP). Individuals in ESP that overlapped with individuals from the CHARGE effort (n = 40) were omitted to avoid duplicate individuals in analyses. Institutional Review Boards or Ethics Committees approved each contributing study. All participants provided written informed consent to participate in genetic research on cardiovascular disease.

### Exome sequencing

We performed a combined analysis of exome sequencing conducted in the CHARGE consortium [27] and ESP [26]. In CHARGE, the exome was captured using NimbleGen SeqCap EZ VCRome (Roche, Basel, Switzerland). The enriched library was then sequenced by Illumina HiSeq platform at Human Genome Sequencing Center at Baylor College of Medicine. The Mercury pipeline [28] was used to process sequencing data, whereas the raw short reads were aligned to the reference human genome (NCBI Genome Build 37, 2009) by Burrows-Wheeler Aligner [29], and the variants were called by Atlas [30]. The mean read depth was 92x, and more than 92% of target regions were covered by at least 20 unique reads. Rigorous quality control was performed to exclude low-quality variants or samples. We excluded variants that were multi-allelic or monomorphic, had a missing rate higher than 20%, had mean depth higher than 500, or had Hardy-Weinberg equilibrium p-value less than 5x10^-6^ within ancestry groups. For individual samples, we calculated four quality metrics: mean depth, transition to transversion (Ti/Tv) ratio, number of singletons, and heterozygote to homozygote ratio. Samples with any metric exceeding 6 standard deviations in the respective study were omitted from analyses.

ESP included samples from 6823 individuals of European or African ancestry. The details of library construction, sequencing and alignment have been described previously [31–33]. Briefly, the exome was captured using either Agilent SureSelect Human All Exon 50Mb (Agilent, Santa Clara, CA) or NimbleGen SeqCap EZ VCRome (Roche, Basel, Switzerland). The sequencing was performed at the University of Washington and at the Broad Institute of MIT and Harvard. The mean depth was 127x. Variants with mean depth greater than 500, or with missing rate greater than 20% were excluded.

### AF ascertainment

Ascertainment of AF in each cohort has been described previously [15]. Briefly, ascertainment of AF was standardized at each participating study and included the presence of either atrial fibrillation or flutter observed on a study electrocardiogram, within obtained medical encounters, or indicated by billing codes. Both incident and prevalent AF were treated together as AF cases for the purposes of this analysis. For ESP, AF information was obtained from the phenotype file (“ESP6800_Phenotype_Update_061212_final.xlsx”), from which individual level phenotypic data was provided.

### Statistical analyses

Each cohort from CHARGE performed separate analyses and shared results for downstream meta-analysis. For ESP, samples from all cohorts were treated as a single sample for analyses, and adjusted for study sites and capture kits.

For common variants with minor allele frequency (MAF) at least 1%, the association of variants with AF was tested by multivariable logistic regression (ARIC, CHS, and ESP) or logistic generalized estimating equation to account for familial correlation (FHS). In common variant association analyses, we also included noncoding variants in regions flanking exons that were captured by the exome arrays. For rare variants (MAF<1%), we pooled all rare variants based on RefSeq gene regions, and jointly tested their associations with AF with the Sequence Kernel Association Test (SKAT) [34]. To circumvent the dilution of signals by variants with unknown functions, our primary analysis of rare variants focused on nonsynonymous and splice-site variants. In secondary analyses, we limited the analysis to damaging variants, defined as nonsense variants or variants predicted to be damaging by PolyPhen [35] or SIFT [36].

For both common and rare variant analyses, models adjusted for age and sex, and stratified by ancestry (European or African American). ARIC and CHS additionally adjusted for their clinical sites, FHS accounted for family structure. The association analyses were performed using the R package seqMeta (http://cran.r-project.org/web/packages/seqMeta/). Each cohort provided single variant score tests as well as genotype covariance matrices for all variants. We meta-analyzed the individual-cohort results using the inverse-variance weighted fixed effects model in seqMeta. Bonferroni correction was used to adjust for multiple testing, and the significance was defined as 0.05/N, where N is the total number of tests.

### Pathway analyses

Pathway analyses were used to investigate the collective effects of multiple genetic variants on AF risk. Each common variant was assigned a score to indicate its association with AF. The score was calculated as –log_10_(*P*-value), where the *P*-value was derived from the common variant test described above. The genetic variant was then mapped back to RefSeq genes (August 23, 2015). A gene score was defined as the highest score of variants within 110kb upstream and 40kb downstream of the gene’s most extreme transcript boundaries, which was anticipated to include the majority of *cis-*regulatory gene elements [37]. For rare variants, each gene was assigned a score equivalent to –log_10_(*P*-value), in which the *P*-value was derived from the SKAT test described previously.

We examined the enrichment of AF-related variants in an AF gene set comprised of 37 genes previously implicated in AF (S1 Table). Genes identified on the basis of GWAS results were selected on the basis of proximity to the AF susceptibility signal, biological literature supporting a putative functional role in AF pathogenesis, or using GRAIL [38]. Gene set enrichment analysis [39] was used to estimate the enrichment, and the significant gene sets were defined as those with *P*-value less than 0.05/3 = 0.017.

## Supporting Information

S1 Table Genes previously implicated in atrial fibrillation pathogenesis. (DOCX)

S2 Table Average sequencing coverage among atrial fibrillation genes. (DOCX)

S3 Table Conditional analysis of a previously discovered noncoding variant at chromosome 10q22 and a newly discovered coding variant within *SYNPO2L*. (DOCX)

S4 Table Ten most significantly associated genes with atrial fibrillation, based on analyses of rare nonsynonymous or splice variants. (DOCX)

S5 Table Ten most significantly associated genes with atrial fibrillation, based on analyses of rare damaging variants. (DOCX)

S6 Table Ten most significantly associated genes with atrial fibrillation, based on analyses of all rare variants. (DOCX)

S7 Table Extended list of investigators that participated in the NHLBI GO Exome Sequencing Project. (DOCX)

S1 Fig QQ plot for common variant association analysis. (DOCX)
