# Supplementary material for: Whole Exome Sequencing in Atrial Fibrillation
Source: PLoS Genet. 2016 Sep 2;12(9):e1006284. doi: 10.1371/journal.pgen.1006284 (PMC5010214; doi:10.1371/journal.pgen.1006284)
Supplement: S1 Table — (DOCX) [file pgen.1006284.s001.docx]

**Supplemental Table 1.** Genes previously implicated in atrial fibrillation pathogenesis.

| **Gene** | **Reference** | **Gene** | **Reference** |
| --- | --- | --- | --- |
| *ACE* | [1] | *KCNN3* | [2] |
| *AGT* | [3, 4] | *KCNQ1* | [5-7] |
| *ANK2* | [8] | *LMNA* | [9, 10] |
| *C9ORF3* | [2] | *MYOZ1* | [2] |
| *CAND2* | [11] | *NEURL1* | [2] |
| *CAV1* | [2] | *NPPA* | [12] |
| *CAV3* | [2] | *NUP155* | [13] |
| *GATA4* | [14-17] | *PITX2* | [2] |
| *GATA5* | [18, 19] | *PRRX1* | [2] |
| *GATA6* | [20, 21] | *SCN10A* | [22] |
| *GJA1* | [11] | *SCN1B* | [23, 24] |
| *GJA5* | [25, 26] | *SCN2B* | [23] |
| *HCN4* | [2] | *SCN3B* | [27] |
| *IL6R* | [28] | *SCN5A* | [29-35] |
| *KCNA5* | [36] | *SYNE2* | [2] |
| *KCNE1* | [37-39] | *SYNPO2L* | [2] |
| *KCNE2* | [40] | *TBX5* | [11, 41] |
| *KCNH2* | [42, 43] | *ZFHX3* | [2] |
| *KCNJ2* | [44] |  |  |

1. Bedi M, McNamara D, London B, Schwartzman D. Genetic susceptibility to atrial fibrillation in patients with congestive heart failure. Heart Rhythm. 2006;3(7):808-12. PubMed PMID: 16818212.

2. Ellinor PT, Lunetta KL, Albert CM, Glazer NL, Ritchie MD, Smith AV, et al. Meta-analysis identifies six new susceptibility loci for atrial fibrillation. Nat Genet. 2012;44(6):670-5. Epub 2012/05/01. doi: 10.1038/ng.2261. PubMed PMID: 22544366; PubMed Central PMCID: PMC3366038.

3. Tsai CT, Lai LP, Lin JL, Chiang FT, Hwang JJ, Ritchie MD, et al. Renin-angiotensin system gene polymorphisms and atrial fibrillation. Circulation. 2004;109(13):1640-6. PubMed PMID: 15023884.

4. Wang QS, Li YG, Chen XD, Yu JF, Wang J, Sun J, et al. Angiotensinogen polymorphisms and acquired atrial fibrillation in Chinese. J Electrocardiol. 2009. Epub 2009/11/26. doi: S0022-0736(09)00537-8 [pii]

10.1016/j.jelectrocard.2009.09.009. PubMed PMID: 19932491.

5. Chen YH, Xu SJ, Bendahhou S, Wang XL, Wang Y, Xu WY, et al. KCNQ1 gain-of-function mutation in familial atrial fibrillation. Science. 2003;299(5604):251-4. PubMed PMID: 12522251.

6. Otway R, Vandenberg JI, Guo G, Varghese A, Castro ML, Liu J, et al. Stretch-sensitive KCNQ1 mutation A link between genetic and environmental factors in the pathogenesis of atrial fibrillation? J Am Coll Cardiol. 2007;49(5):578-86. PubMed PMID: 17276182.

7. Das S, Makino S, Melman YF, Shea MA, Goyal SB, Rosenzweig A, et al. Mutation in the S3 segment of KCNQ1 results in familial lone atrial fibrillation. Heart Rhythm. 2009;6(8):1146-53. Epub 2009/07/28. doi: S1547-5271(09)00439-1 [pii]

10.1016/j.hrthm.2009.04.015. PubMed PMID: 19632626.

8. Cunha SR, Hund TJ, Hashemi S, Voigt N, Li N, Wright P, et al. Defects in ankyrin-based membrane protein targeting pathways underlie atrial fibrillation. Circulation. 2011;124(11):1212-22. doi: 10.1161/CIRCULATIONAHA.111.023986. PubMed PMID: 21859974; PubMed Central PMCID: PMCPMC3211046.

9. Fatkin D, MacRae C, Sasaki T, Wolff MR, Porcu M, Frenneaux M, et al. Missense mutations in the rod domain of the lamin A/C gene as causes of dilated cardiomyopathy and conduction-system disease. N Engl J Med. 1999;341(23):1715-24. PubMed PMID: 10580070.

10. Sebillon P, Bouchier C, Bidot LD, Bonne G, Ahamed K, Charron P, et al. Expanding the phenotype of LMNA mutations in dilated cardiomyopathy and functional consequences of these mutations. J Med Genet. 2003;40(8):560-7. Epub 2003/08/16. PubMed PMID: 12920062.

11. Sinner MF, Tucker NR, Lunetta KL, Ozaki K, Smith JG, Trompet S, et al. Integrating Genetic, Transcriptional, and Functional Analyses to Identify Five Novel Genes for Atrial Fibrillation. Circulation. 2014. doi: 10.1161/CIRCULATIONAHA.114.009892. PubMed PMID: 25124494.

12. Hodgson-Zingman DM, Karst ML, Zingman LV, Heublein DM, Darbar D, Herron KJ, et al. Atrial natriuretic peptide frameshift mutation in familial atrial fibrillation. N Engl J Med. 2008;359(2):158-65. Epub 2008/07/11. doi: 359/2/158 [pii]

10.1056/NEJMoa0706300. PubMed PMID: 18614783.

13. Zhang X, Chen S, Yoo S, Chakrabarti S, Zhang T, Ke T, et al. Mutation in nuclear pore component NUP155 leads to atrial fibrillation and early sudden cardiac death. Cell. 2008;135(6):1017-27. Epub 2008/12/17. doi: 10.1016/j.cell.2008.10.022. PubMed PMID: 19070573.

14. Posch MG, Boldt LH, Polotzki M, Richter S, Rolf S, Perrot A, et al. Mutations in the cardiac transcription factor GATA4 in patients with lone atrial fibrillation. European journal of medical genetics. 2010;53(4):201-3. doi: 10.1016/j.ejmg.2010.03.008. PubMed PMID: 20363377.

15. Yang YQ, Wang MY, Zhang XL, Tan HW, Shi HF, Jiang WF, et al. GATA4 loss-of-function mutations in familial atrial fibrillation. Clin Chim Acta. 2011;412(19-20):1825-30. doi: 10.1016/j.cca.2011.06.017. PubMed PMID: 21708142.

16. Jiang JQ, Shen FF, Fang WY, Liu X, Yang YQ. Novel GATA4 mutations in lone atrial fibrillation. Int J Mol Med. 2011;28(6):1025-32. doi: 10.3892/ijmm.2011.783. PubMed PMID: 21874226.

17. Wang J, Sun YM, Yang YQ. Mutation spectrum of the GATA4 gene in patients with idiopathic atrial fibrillation. Molecular biology reports. 2012;39(8):8127-35. doi: 10.1007/s11033-012-1660-6. PubMed PMID: 22552926.

18. Yang YQ, Wang J, Wang XH, Wang Q, Tan HW, Zhang M, et al. Mutational spectrum of the GATA5 gene associated with familial atrial fibrillation. Int J Cardiol. 2012;157(2):305-7. doi: 10.1016/j.ijcard.2012.03.132. PubMed PMID: 22483626.

19. Wang XH, Huang CX, Wang Q, Li RG, Xu YJ, Liu X, et al. A novel GATA5 loss-of-function mutation underlies lone atrial fibrillation. Int J Mol Med. 2013;31(1):43-50. doi: 10.3892/ijmm.2012.1189. PubMed PMID: 23175127.

20. Yang YQ, Li L, Wang J, Zhang XL, Li RG, Xu YJ, et al. GATA6 loss-of-function mutation in atrial fibrillation. European journal of medical genetics. 2012;55(10):520-6. doi: 10.1016/j.ejmg.2012.06.007. PubMed PMID: 22750565.

21. Li J, Liu WD, Yang ZL, Yang YQ. Novel GATA6 loss-of-function mutation responsible for familial atrial fibrillation. Int J Mol Med. 2012;30(4):783-90. doi: 10.3892/ijmm.2012.1068. PubMed PMID: 22824924.

22. Jabbari J, Olesen MS, Yuan L, Nielsen JB, Liang B, Macri V, et al. Common and rare variants in SCN10A modulate the risk of atrial fibrillation. Circ Cardiovasc Genet. 2015;8(1):64-73. doi: 10.1161/HCG.0000000000000022. PubMed PMID: 25691686; PubMed Central PMCID: PMC4392342.

23. Watanabe H, Darbar D, Kaiser DW, Jiramongkolchai K, Chopra S, Donahue BS, et al. Mutations in sodium channel beta1- and beta2-subunits associated with atrial fibrillation. Circ Arrhythm Electrophysiol. 2009;2(3):268-75. Epub 2009/10/08. doi: CIRCEP.108.779181 [pii]

10.1161/CIRCEP.108.779181. PubMed PMID: 19808477.

24. Hayashi K, Konno T, Tada H, Tani S, Liu L, Fujino N, et al. Functional Characterization of Rare Variants Implicated in Susceptibility to Lone Atrial Fibrillation. Circ Arrhythm Electrophysiol. 2015;8(5):1095-104. doi: 10.1161/CIRCEP.114.002519. PubMed PMID: 26129877.

25. Gollob MH, Jones DL, Krahn AD, Danis L, Gong XQ, Shao Q, et al. Somatic mutations in the connexin 40 gene (GJA5) in atrial fibrillation. N Engl J Med. 2006;354(25):2677-88. PubMed PMID: 16790700.

26. Firouzi M, Ramanna H, Kok B, Jongsma HJ, Koeleman BP, Doevendans PA, et al. Association of human connexin40 gene polymorphisms with atrial vulnerability as a risk factor for idiopathic atrial fibrillation. Circ Res. 2004;95(4):e29-33. PubMed PMID: 15297374.

27. Olesen MS, Jespersen T, Nielsen JB, Liang B, Moller DV, Hedley P, et al. Mutations in sodium channel beta-subunit SCN3B are associated with early-onset lone atrial fibrillation. Cardiovasc Res. 2011;89(4):786-93. doi: 10.1093/cvr/cvq348. PubMed PMID: 21051419.

28. Schnabel RB, Kerr KF, Lubitz SA, Alkylbekova EL, Marcus GM, Sinner MF, et al. Large-scale candidate gene analysis in whites and African Americans identifies IL6R polymorphism in relation to atrial fibrillation: the National Heart, Lung, and Blood Institute's Candidate Gene Association Resource (CARe) project. Circulation Cardiovascular genetics. 2011;4(5):557-64. Epub 2011/08/19. doi: 10.1161/CIRCGENETICS.110.959197. PubMed PMID: 21846873; PubMed Central PMCID: PMC3224824.

29. Darbar D, Kannankeril PJ, Donahue BS, Kucera G, Stubblefield T, Haines JL, et al. Cardiac sodium channel (SCN5A) variants associated with atrial fibrillation. Circulation. 2008;117(15):1927-35. PubMed PMID: 18378609.

30. Ellinor PT, Nam EG, Shea MA, Milan DJ, Ruskin JN, MacRae CA. Cardiac sodium channel mutation in atrial fibrillation. Heart Rhythm. 2008;5(1):99-105. PubMed PMID: 18088563.

31. Benito B, Brugada R, Perich RM, Lizotte E, Cinca J, Mont L, et al. A mutation in the sodium channel is responsible for the association of long QT syndrome and familial atrial fibrillation. Heart Rhythm. 2008;5(10):1434-40. Epub 2008/10/22. doi: S1547-5271(08)00712-1 [pii]

10.1016/j.hrthm.2008.07.013. PubMed PMID: 18929331.

32. Makiyama T, Akao M, Shizuta S, Doi T, Nishiyama K, Oka Y, et al. A novel SCN5A gain-of-function mutation M1875T associated with familial atrial fibrillation. J Am Coll Cardiol. 2008;52(16):1326-34. Epub 2008/10/22. doi: S0735-1097(08)02472-8 [pii]

10.1016/j.jacc.2008.07.013. PubMed PMID: 18929244.

33. McNair WP, Ku L, Taylor MRG, Fain PR, Dao D, Wolfel E, et al. SCN5A mutation associated with dilated cardiomyopathy, conduction disorder, and arrhythmia. Circulation. 2004;110(15):2163-7. doi: 10.1161/01.CIR.0000144458.58660.BB. PubMed PMID: 15466643.

34. Olson TM, Michels VV, Ballew JD, Reyna SP, Karst ML, Herron KJ, et al. Sodium channel mutations and susceptibility to heart failure and atrial fibrillation. JAMA. 2005;293(4):447-54. PubMed PMID: 15671429.

35. Li Q, Huang H, Liu G, Lam K, Rutberg J, Green MS, et al. Gain-of-function mutation of Nav1.5 in atrial fibrillation enhances cellular excitability and lowers the threshold for action potential firing. Biochem Biophys Res Commun. 2009;380(1):132-7. Epub 2009/01/27. doi: S0006-291X(09)00090-4 [pii]

10.1016/j.bbrc.2009.01.052. PubMed PMID: 19167345.

36. Olson TM, Alekseev AE, Liu XK, Park S, Zingman LV, Bienengraeber M, et al. Kv1.5 channelopathy due to KCNA5 loss-of-function mutation causes human atrial fibrillation. Hum Mol Genet. 2006;15(14):2185-91. PubMed PMID: 16772329.

37. Fatini C, Sticchi E, Genuardi M, Sofi F, Gensini F, Gori AM, et al. Analysis of minK and eNOS genes as candidate loci for predisposition to non-valvular atrial fibrillation. Eur Heart J. 2006;27(14):1712-8. PubMed PMID: 16760206.

38. Lai LP, Su MJ, Yeh HM, Lin JL, Chiang FT, Hwang JJ, et al. Association of the human minK gene 38G allele with atrial fibrillation: evidence of possible genetic control on the pathogenesis of atrial fibrillation. Am Heart J. 2002;144(3):485-90. PubMed PMID: 12228786.

39. Prystupa A, Dzida G, Myslinski W, Malaj G, Lorenc T. MinK gene polymorphism in the pathogenesis of lone atrial fibrillation. Kardiol Pol. 2006;64(11):1205-11; discussion 12-3. PubMed PMID: 17165161.

40. Yang Y, Xia M, Jin Q, Bendahhou S, Shi J, Chen Y, et al. Identification of a KCNE2 gain-of-function mutation in patients with familial atrial fibrillation. Am J Hum Genet. 2004;75(5):899-905. PubMed PMID: 15368194.

41. Raychaudhuri S, Plenge RM, Rossin EJ, Ng AC, International Schizophrenia C, Purcell SM, et al. Identifying relationships among genomic disease regions: predicting genes at pathogenic SNP associations and rare deletions. PLoS Genet. 2009;5(6):e1000534. doi: 10.1371/journal.pgen.1000534. PubMed PMID: 19557189; PubMed Central PMCID: PMCPMC2694358.

42. Hong K, Bjerregaard P, Gussak I, Brugada R. Short QT syndrome and atrial fibrillation caused by mutation in KCNH2. J Cardiovasc Electrophysiol. 2005;16(4):394-6. PubMed PMID: 15828882.

43. Sinner MF, Pfeufer A, Akyol M, Beckmann BM, Hinterseer M, Wacker A, et al. The non-synonymous coding IKr-channel variant KCNH2-K897T is associated with atrial fibrillation: results from a systematic candidate gene-based analysis of KCNH2 (HERG). Eur Heart J. 2008;29(7):907-14. Epub 2008/01/29. doi: ehm619 [pii]

10.1093/eurheartj/ehm619. PubMed PMID: 18222980.

44. Xia M, Jin Q, Bendahhou S, He Y, Larroque MM, Chen Y, et al. A Kir2.1 gain-of-function mutation underlies familial atrial fibrillation. Biochem Biophys Res Commun. 2005;332(4):1012-9. PubMed PMID: 15922306.
