# Supplementary material for: Whole Exome Sequencing in Atrial Fibrillation
Source: PLoS Genet. 2016 Sep 2;12(9):e1006284. doi: 10.1371/journal.pgen.1006284 (PMC5010214; doi:10.1371/journal.pgen.1006284)
Supplement: S2 Table — (DOCX) [file pgen.1006284.s002.docx]

**Supplemental Table 2.** Average sequencing fold-coverage among atrial fibrillation genes.

| **Gene** | **Average coverage (CHARGE-S)** | **Average coverage**  **(ESP)** |
| --- | --- | --- |
| *ACE* | 76 | 66 |
| *AGT* | 100 | 89 |
| *ANK2* | 98 | 89 |
| *C9orf3* | 76 | 74 |
| *CAND2* | 95 | 85 |
| *CAV1* | 80 | 79 |
| *CAV3* | 95 | 85 |
| *GATA4* | 79 | 79 |
| *GATA5* | 66 | 56 |
| *GATA6* | 85 | 85 |
| *GJA1* | 83 | 90 |
| *GJA5* | 97 | 89 |
| *HCN4* | 78 | 78 |
| *IL6R* | 96 | 85 |
| *KCNA5* | 82 | 84 |
| *KCNE1* | 78 | 71 |
| *KCNE2* | 78 | 71 |
| *KCNH2* | 76 | 69 |
| *KCNJ2* | 72 | 61 |
| *KCNN3* | 96 | 85 |
| *KCNQ1* | 82 | 84 |
| *LMNA* | 96 | 86 |
| *MYOZ1* | 82 | 86 |
| *NEURL1* | 84 | 88 |
| *NPPA* | 96 | 85 |
| *NUP155* | 95 | 84 |
| *PITX2* | 97 | 89 |
| *PRRX1* | 96 | 85 |
| *SCN10A* | 95 | 86 |
| *SCN1B* | 73 | 72 |
| *SCN2B* | 82 | 87 |
| *SCN3B* | 86 | 88 |
| *SCN5A* | 95 | 86 |
| *SYNE2* | 88 | 82 |
| *SYNPO2L* | 82 | 86 |
| *TBX5* | 78 | 76 |
| *ZFHX3* | 72 | 68 |
