# Supplementary material for: Whole Exome Sequencing in Atrial Fibrillation
Source: PLoS Genet. 2016 Sep 2;12(9):e1006284. doi: 10.1371/journal.pgen.1006284 (PMC5010214; doi:10.1371/journal.pgen.1006284)
Supplement: S3 Table — (DOCX) [file pgen.1006284.s003.docx]

| **Supplemental Table 3**. Conditional analysis of a previously discovered noncoding variant at chromosome 10q22 and a newly discovered coding variant within *SYNPO2L*. | | | | | | |
| --- | --- | --- | --- | --- | --- | --- |
|  |  |  | **Single SNP analysis** | | **Conditional analysis** | |
| **SNP** | **Position (HG19)** | **Variant description** | **OR (95% CI)** | **P** | **OR (95% CI)** | **P** |
| rs10824026* | 75421208 | Intergenic, upstream of *SYNPO2L* | 1.09 (1.02-1.16) | 0.01 | 1.01 (0.83-1.23) | 0.89 |
| rs3812629 | 75407290 | *SYNPO2L* p.Pro707Leu | 0.92 (0.86-0.98) | 9.0x10^-3^ | 0.94 (0.77-1.15) | 0.53 |
| * Top SNP at chromosome 10q22 from prior meta-analysis.[2]  Analyses adjusted for age, sex, and site (ARIC) or clinic (CHS) and meta-analyzed using a fixed effects inverse variance weighted method. Analyses included 671 individuals from CHS, 6360 from ARIC, and 1256 from FHS of European ancestry. | | | | | | |
