# Supplementary material for: Whole Exome Sequencing in Atrial Fibrillation
Source: PLoS Genet. 2016 Sep 2;12(9):e1006284. doi: 10.1371/journal.pgen.1006284 (PMC5010214; doi:10.1371/journal.pgen.1006284)
Supplement: S4 Table — (DOCX) [file pgen.1006284.s004.docx]

**Supplemental Table 4.** Ten most significantly associated genes with atrial fibrillation, based on analyses of rare nonsynoymous or splice variants.

| **Gene** | **P** | **Qmeta** | **CMAF** | **No. SNPs** |
| --- | --- | --- | --- | --- |
| *IL17REL* | 1.3x10^-5^ | 293565.5 | 0.021 | 34 |
| *OR10G7* | 1.6x10^-5^ | 96026.06 | 0.022 | 19 |
| *ACY3* | 1.8x10^-5^ | 159160.1 | 0.017 | 42 |
| *AMPD1* | 6.7x10^-5^ | 93026.86 | 0.021 | 72 |
| *GDF9* | 3.1x10^-4^ | 55825.15 | 0.014 | 38 |
| *TUSC3* | 4.0x10^-4^ | 97024.18 | 0.011 | 26 |
| *EGFL8* | 4.3x10^-4^ | 74878.52 | 0.012 | 33 |
| *IPO4* | 5.5x10^-4^ | 126250.4 | 0.031 | 93 |
| *IQCF5* | 9.0x10^-4^ | 94247.42 | 0.011 | 21 |
| *HRC* | 9.4x10^-4^ | 146779.9 | 0.037 | 54 |
