# Supplementary material for: Whole Exome Sequencing in Atrial Fibrillation
Source: PLoS Genet. 2016 Sep 2;12(9):e1006284. doi: 10.1371/journal.pgen.1006284 (PMC5010214; doi:10.1371/journal.pgen.1006284)
Supplement: S5 Table — (DOCX) [file pgen.1006284.s005.docx]

**Supplemental Table 5.** Ten most significantly associated genes with atrial fibrillation, based on analyses of rare damaging variants.

| **Gene** | **P** | **Qmeta** | **CMAF** | **No. SNPs** |
| --- | --- | --- | --- | --- |
| *IL17REL* | 1.9x10^-6^ | 206466.5 | 0.012 | 23 |
| *AMPD1* | 6.9x10^-5^ | 90954.44 | 0.020 | 64 |
| *CDC42BPG* | 2.0x10^-4^ | 100458.1 | 0.026 | 105 |
| *GDF9* | 2.9x10^-4^ | 55236.88 | 0.013 | 30 |
| *CCDC144NL* | 3.9x10^-4^ | 58908.88 | 0.011 | 33 |
| *TUSC3* | 4.2x10^-4^ | 95331.6 | 0.010 | 25 |
| *CHRDL2* | 5.9x10^-4^ | 91676.82 | 0.017 | 52 |
| *ACY3* | 7.7x10^-4^ | 76721.27 | 0.013 | 32 |
| *LRPAP1* | 1.2x10^-4^ | 72649.21 | 0.014 | 33 |
| *ABCD4* | 1.4x10^-3^ | 95223.86 | 0.026 | 81 |
