# Supplementary material for: Whole Exome Sequencing in Atrial Fibrillation
Source: PLoS Genet. 2016 Sep 2;12(9):e1006284. doi: 10.1371/journal.pgen.1006284 (PMC5010214; doi:10.1371/journal.pgen.1006284)
Supplement: S6 Table — (DOCX) [file pgen.1006284.s006.docx]

**Supplemental Table 6.** Ten most significantly associated genes with atrial fibrillation, based on analyses of all rare variants.

| **Gene** | **P** | **Qmeta** | **CMAF** | **No. SNPs** |
| --- | --- | --- | --- | --- |
| *ACY3* | 2.2x10^-7^ | 296441.5 | 0.053 | 72 |
| *IL17REL* | 1.3x10^-5^ | 300550.8 | 0.036 | 62 |
| *OR10G7* | 2.3x10^-5^ | 96819.48 | 0.025 | 26 |
| *IRF9* | 5.8x10^-5^ | 171301.4 | 0.040 | 61 |
| *PPIAL4A* | 1.0x10^-4^ | 2154.739 | 0.001 | 1 |
| *MIR216A* | 1.3x10^-4^ | 6318.234 | 0.001 | 4 |
| *YIPF1* | 1.4x10^-4^ | 50050.87 | 0.014 | 64 |
| *TLK2* | 1.8x10^-4^ | 18051.19 | 0.013 | 45 |
| *C4A* | 1.9x10^-4^ | 9076.575 | 0.005 | 8 |
| *FUT3* | 2.0x10^-4^ | 120379.5 | 0.023 | 55 |
