# Supplementary material for: Whole Exome Sequencing in Atrial Fibrillation
Source: PLoS Genet. 2016 Sep 2;12(9):e1006284. doi: 10.1371/journal.pgen.1006284 (PMC5010214; doi:10.1371/journal.pgen.1006284)
Supplement: S1 Fig — (DOCX) [file pgen.1006284.s008.docx]

**Supplemental Figure.** QQ plot for common variant association analysis from both CHARGE cohorts (ARIC, CHS and FHS) and ESP.

**
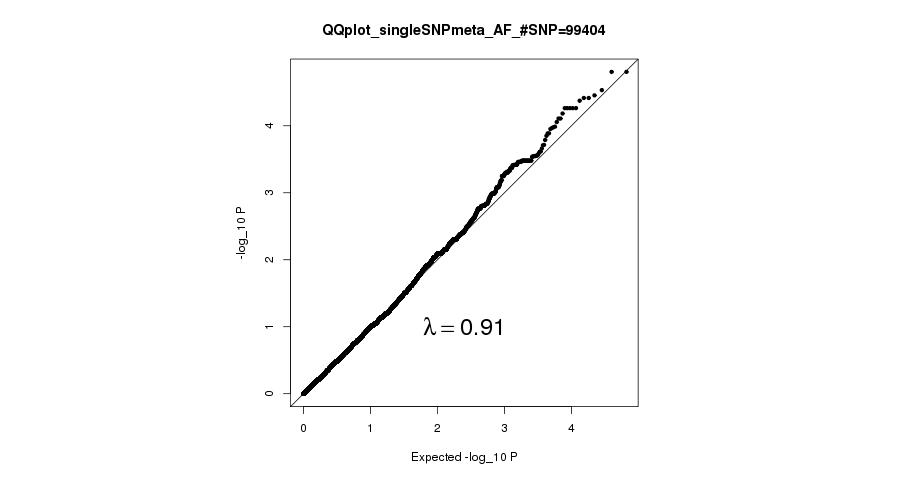
**
